# Whole-Genome Sequence of Mucilaginibacter jinjuensis Strain KACC 16571^T^

**DOI:** 10.1128/mra.00269-23

**Published:** 2023-05-03

**Authors:** Hyorim Choi, Seunghwan Kim, Yiseul Kim, Daseul Lee, Byeong-Hak Han, Seung-Beom Hong, Soon-Wo Kwon, Yong Hoon Lee, Jun Heo

**Affiliations:** a Agricultural Microbiology Division, National Institute of Agricultural Sciences, Rural Development Administration, Wanju-gun, Jeollabuk-do, Republic of Korea; b Division of Biotechnology, Chonbuk National University, Iksan, Republic of Korea; DOE Joint Genome Institute

## Abstract

We report the whole-genome sequence of Mucilaginibacter jinjuensis type strain KACC 16571, which was isolated from rotten wood in South Korea. The genome of Mucilaginibacter jinjuensis KACC 16571^T^ consists of a 6.16-Mb circular chromosome, with a G+C content of 42.1% and 5,262 total predicted coding genes.

## ANNOUNCEMENT

The genus *Mucilaginibacter* was first reported in 2007 (1), and 79 species have been reported thus far. These strains are Gram-negative, non-spore forming, nonmotile, rod-shaped bacteria with carboxymethyl cellulose (CM-cellulose) and xylan degradation activity (2). Mucilaginibacter jinjuensis KACC 16571^T^ was isolated from rotten wood in 2010. It has a pale orange pigment and the ability to degrade xylan (3). This strain was deposited at the Korean Agricultural Culture Collection (KACC) in 2011 and reported as a novel species in 2013 (3). However, genome analysis of *M. jinjuensis* has not been reported until now.

Here, we report the whole-genome sequence of Mucilaginibacter jinjuensis KACC 16571^T^, isolated in Jinju-si, South Korea (3). The authors deposited this strain at the Korean Agricultural Culture Collection (KACC), from which we obtained it. The strain was subcultured on Reasoner’s 2A (R2A) medium (BD Difco, NJ, USA), pH 6.0, at 30°C for 2 days under aerobic conditions. Genomic DNA extraction was performed using the MagAttract high-molecular-weight (HMW) DNA kit (Qiagen, Hilden, Germany) according to the manufacturer’s instructions. Using the same DNA products, genome sequence analysis was conducted using the Illumina NovaSeq 6000 (Illumina, CA, USA) and PacBio Sequel IIe (Pacific Biosciences, CA, USA) platforms. Genomic DNA libraries were generated using the TruSeq Nano DNA high-throughput library prep kit (Illumina). For template preparation using the PacBio SMRTbell prep kit 3.0, libraries (total, 10 μL) were prepared and analyzed with the Sequel II Bind kit 3.2 and Int Ctrl 3.2. Sequencing was performed using the Sequel II sequencing kit 2.0 and single-molecule real-time (SMRT) cell 8M trays. High-fidelity (Hi-Fi) reads were produced using the PacBio Sequel IIe system. The microbial assembly application SMRTLink 11.0.0.146107 was used for assembly based on the Hierarchical Genome Assembly Process (HGAP) (4), and the assembly based on long-read data was then revised using the Illumina reads to correct errors and improve accuracy with Pilon 1.21 (5). Gene prediction and annotation were performed using the NCBI Prokaryotic Genome Annotation Pipeline 6.4 (6). All tools were run with default parameters unless otherwise specified.

The Illumina NovaSeq 6000 and PacBio Sequel IIe sequencing generated 14,614,268 read pairs (2 × 150 bp) and 122,630 long reads (1,140,048,571 bp; *N*_50_, 9,906 bp), respectively. The genome of *M. jinjuensis* KACC 16571 consists of a single circular chromosome (6,157,393 bp; G+C content, 42.1%) and no plasmids. The contig assembly contains 5,262 total coding sequences, 9 rRNA genes, 54 tRNA genes, 3 noncoding RNA genes, and 12 pseudogenes.

### Data availability.

The complete genome sequence of Mucilaginibacter jinjuensis KACC 16571^T^ was deposited at NCBI GenBank under the BioProject accession number PRJNA930584, the BioSample accession number SAMN33013692, and the GenBank accession number CP117167.1. The raw Illumina and PacBio reads can be found under the SRA accession numbers SRR23796428 and SRR23952319, respectively.
